# Migratory Connectivity at High Latitudes: Sabine’s Gulls (*Xema sabini*) from a Colony in the Canadian High Arctic Migrate to Different Oceans

**DOI:** 10.1371/journal.pone.0166043

**Published:** 2016-12-14

**Authors:** Shanti E. Davis, Mark Maftei, Mark L. Mallory

**Affiliations:** 1 High Arctic Gull Research Group, Victoria, British Columbia, Canada; 2 Department of Biology, Memorial University of Newfoundland, St. John’s, Newfoundland and Labrador, Canada; 3 Department of Biology, Acadia University, Wolfville, Nova Scotia, Canada; Liverpool John Moores University, UNITED KINGDOM

## Abstract

The world's Arctic latitudes are some of the most recently colonized by birds, and an understanding of the migratory connectivity of circumpolar species offers insights into the mechanisms of range expansion and speciation. Migratory divides exist for many birds, however for many taxa it is unclear where such boundaries lie, and to what extent these affect the connectivity of species breeding across their ranges. Sabine’s gulls (*Xema sabini*) have a patchy, circumpolar breeding distribution and overwinter in two ecologically similar areas in different ocean basins: the Humboldt Current off the coast of Peru in the Pacific, and the Benguela Current off the coasts of South Africa and Namibia in the Atlantic. We used geolocators to track Sabine’s gulls breeding at a colony in the Canadian High Arctic to determine their migratory pathways and wintering sites. Our study provides evidence that birds from this breeding site disperse to both the Pacific and Atlantic oceans during the non-breeding season, which suggests that a migratory divide for this species exists in the Nearctic. Remarkably, members of one mated pair wintered in opposite oceans. Our results ultimately suggest that colonization of favorable breeding habitat may be one of the strongest drivers of range expansion in the High Arctic.

## Introduction

Determining the extent to which breeding populations overlap during the non-breeding season (i.e., migratory connectivity) is essential to interpret the ecological and evolutionary patterns of migratory species [1]. Migratory divides delineate the boundaries between adjacent breeding populations with divergent migration pathways and are common in many migratory bird species [2–4]. Intraspecific variation in migratory routes may be driven by physical factors such as past glacial events, geographical barriers, or suitable habitat for refueling [5–7], or biological factors such as the distribution of resources, energetic costs of migration, or competition between breeding populations [8,9].

The Canadian High Arctic is a vast archipelago which forms part of a nearly continuous area of relatively homogenous High Arctic tundra habitat extending from the Nearctic to the Palearctic [10]. Even species which breed across large or even circumpolar ranges within this region are typically divided into discrete populations that breed and winter in disjunct ranges with varying degrees of migratory connectivity [10]. The study of migration patterns in the Canadian High Arctic is of particular interest for several reasons: (i) it is ecologically a very “young” area, having only become accessible as nesting habitat for birds since the last major ice age [5]; (ii) it extends so far north of the Nearctic continental landmass that in its northern reaches it is geographically an equally likely destination for migrants from the both the Nearctic and western Palearctic; and (iii) it extends from the North American continent symmetrically, so that its relative midpoint lies approximately equidistant from both the Atlantic and Pacific coasts [11]. These factors have led to the colonization of the Canadian High Arctic archipelago by migratory seabird species from three source regions: Atlantic, Pacific and Palearctic [12–14]. Determining how species and populations are distributed through the Canadian High Arctic archipelago can help clarify the evolutionary process behind the migration patterns seen in Arctic birds as a group [11].

For most Palearctic migratory birds, there is a distinct migratory divide at 100° E along the Taymyr Peninsula in Russia, which forms the most northerly continental barrier to east-west migration, and lies roughly halfway between suitable wintering habitat in the Atlantic and Pacific regions [15,16].

Efforts to study migration patterns in the Nearctic have failed to find a corresponding geographic divide between migratory bird species [11]. For example, many shorebirds appear to be divided in the western Arctic [13], while some passerines follow a divide in the east [17]. Jaegers, terns, and gulls [11], as well as some waterfowl [12,18] migrate both east and west out of the Nearctic, with no consistent shared geographic boundary across species. It remains unclear exactly what factors result in these inconsistencies, but the relatively recent colonization of the region as a whole may be an important factor.

The Sabine’s gull (*Xema sabini*) is a small seabird that exhibits a patchy, circumpolar breeding range [19]. It is highly pelagic in the non-breeding season, and spends the majority of its annual cycle in offshore waters [20]. All breeding populations are presumed to migrate to either of two known wintering areas in major upwelling systems in the southern hemisphere [20,21]. The Pacific wintering population occupies a region within the Humboldt Current off the coast of Peru [22], while the Atlantic wintering population occupies a region within the Benguela Current off the coast of South Africa and Namibia [20,23]. It remains unclear how Sabine's gulls segregate between these two ecologically similar but geographically disparate wintering areas, and the distribution of Atlantic and Pacific wintering birds at breeding colonies is unknown [19,24]. Birds breeding in Siberia, Alaska, and the Western Canadian Arctic are thought to winter in the Pacific, while birds from breeding sites in the Eastern Canadian Arctic, Greenland, and Svalbard are thought to winter in the Atlantic [21]. The migratory divide between Atlantic and Pacific wintering populations in the Palearctic is thought to lie along the Taymyr Peninsula [15,16], while the divide in the Nearctic is presumed to lie somewhere in the central Canadian Arctic [21,25].

Here, we used geolocators to track Sabine’s gulls breeding at a colony in the central Canadian High Arctic to determine their migratory pathways and wintering sites. We interpret the revealed migratory patterns of Sabine’s gulls from this site in relation to the ecology and evolution of Arctic breeding migratory birds.

## Methods

### Ethics Statement

All work was conducted under valid permits (CWS Animal Care EC-PN-11-020, CWS Scientific Permit NUN-SCI-09-01, Government of Nunavut Wildlife Research Licence WL 2010–042, Nunavut Water Board licence 3BC-TER0811, Indian and Northern Affairs Land Use Reserve 068H16001, and CWS Banding Permit 10694), and their renewals.

### Study Site

**Fig 1:**
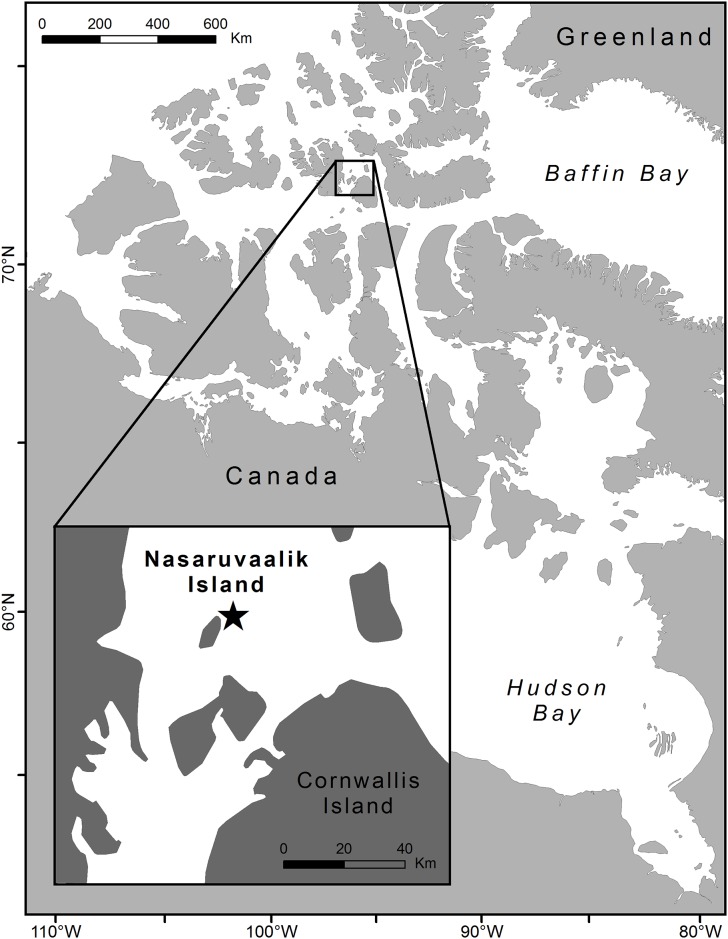
Location of the study site at Nasaruvaalik Island, Nunavut (75.8° N, 96.3° W), in the Canadian High Arctic.

We conducted field research on Nasaruvaalik Island, Nunavut, (75.8° N, 96.3° W; Fig 1), between early June and late August over five years between 2008–2012. Nasaruvaalik Island is a small gravel island 1.4 km^2^ in size, supporting a large and diverse colony of marine birds that forage in several nearby polynyas. The island is characteristic of the High Arctic tundra ecoregion [26] and has been previously described in detail [27]. Sabine’s gulls are annual breeders, and we have recorded 16–31 breeding pairs annually over eight years of study, all of which nest in association with both Arctic terns (*Sterna paradisaea*) and Ross’s gulls (*Rhodostethia rosea*) in two colonies at either end of the island. Nesting habitat in the colonies consists of low gravel beach ridges interspersed with patches of moss and purple saxifrage (*Saxifraga oppositifolia*) and small, shallow ponds [27]. Sabine’s gull philopatry at this site is high (mean annual return rate of 80% over 6 years), based on capture-mark-resight data (S. E. Davis, unpubl. data).

### Deployment and Recovery of Geolocators

We deployed 47 geolocators (44 LAT2900 and 3 LAT2500, Lotek Wireless, Canada) on 33 adult breeding Sabine gulls on Nasaruvallik Island over three years. In 2008, we deployed geolocators on three birds. In 2010, we deployed geolocators on 23 birds, one of which was previously tagged in 2008. In 2011, we deployed geolocators on 21 birds, 13 of which were tagged previously in 2010. In total, we deployed geolocators on 16 females and 17 males, 14 of which (seven males and seven females) we tagged twice. We captured breeding Sabine’s gulls with a spring-loaded bow net [28] or a handheld CO_2_ powered net gun (see [29] for details). We attached geolocators to Darvic tarsal bands with plastic cable ties, totaling 2.1g (LAT2900) and 3.8g (LAT2500), averaging 1.1% and 2.0% of adult body weight, respectively. All tagged birds were also fitted with a numbered metal band and a unique combination of colored Darvic bands on the opposite leg. We determined the sex of tagged birds through an analysis of 2–3 drops of blood collected from the brachial vein. We recaptured tagged birds the following year to recover the geolocators (one unit was recovered after two years), and downloaded the data in LAT Viewer Studio (Lotek Wireless, Canada).

### Data Processing

The geolocators used in this study estimated location once daily; latitude was estimated from the duration of daylight between sunset and sunrise, and longitude from the exact time of sunrise and sunset [30]. The geolocators sampled sea-surface temperature (SST) when immersed for more than two consecutive samples (i.e., 120 s) and recorded the minimum daily value (°C) [31]. To improve the accuracy of latitude estimates, we used SST correlation (LAT Viewer Studio) based on the approach used by Shaffer et al. [32], which allowed us to retain data around the equinoxes. We used 8-day composites of nighttime SST grids from the MODIS TERRA satellite in this study (http://whiteshark.stanford.edu/public/lotek_sst/, 4 km resolution), which are suitable for comparison to the tag values [33]. We then filtered locations [34] to remove positions implying an unrealistic flight speed in Program R [35]. We assumed Sabine's gulls did not exceed a maximum velocity of 13.9 m/s (> 50 km/h sustained over a 48 h period) [36]. To further reduce the mean error in positions estimates, we smoothed each track using a moving weighted average (with a window size of three), whereby each smoothed position was the weighted average (in a 1:2:1 ratio) of the previous, current, and subsequent position (as per [37]). Fixed start positions (at breeding colony) and positions that showed large daily movements (greater than 4° of longitude or 6° degrees of latitude) were not smoothed to avoid introducing positional errors [38].

### Analysis of Movement Data

We pooled all valid locations and generated kernel density estimations to represent the annual distribution of tracked birds (ESRI ArcGIS 10.1, search radius: 200 km, output cell size: 10 km). A search radius of 200 km was chosen for analysis in this study in order to be directly comparable to recent studies of arctic breeding long distance migrants [20,39]. We created occupancy contours (25, 50, 75%) in Geospatial Modelling Environment (GME; [40]) to determine areas of high use throughout the annual cycle. We used the 50% occupancy contour generated around either one of the known wintering areas in the Southern Hemisphere [19,20] to set the boundary for the “wintering area” (as per [37]). For the purpose of this study, we did not use positions that occurred after the wintering period (spring migration) in the remaining analysis.

We assigned positions to either “stopover” or “travel” categories with each bird initially defined to be in a stopover period (i.e., starting at the breeding site). We identified transition to a travel period when three or more positions (within a sliding window of five) showed movement more than 100 km/d, which represents the mean daily movement during the wintering period. Similarly, we identified transition back to a stopover period when three or more positions failed to meet the distance criteria (less than 100 km/d). This approach is comparable to methods used by similar studies of migratory seabirds breeding in the arctic [37,39], where distance between daily positions is used to reduce bias towards the poles when using change in longitude [41] and bias towards east-west migration when using change in latitude [20,42]. Stopover periods were then examined for burst travel days, which occurred when birds travelled fast and far for 1–2 d, which would not trigger a transition to travel, however birds were clearly travelling to a new stopover area [33]. These burst travel days were manually adjusted to reflect the travel behavior.

Tracks were then split into two periods; fall migration and winter. Fall migration was defined as the period between departure from the breeding area (i.e., first “travel” location identified after breeding period) and arrival to the wintering area (i.e., first “stopover” location within the pre-defined wintering area) (as per [37,43]). For each wintering site (Pacific and Atlantic), we generated kernel density estimations (ESRI ArcGIS 10.1, search radius 200 km, output cell size 10 km) using winter locations, which were first transformed to an equal area projection appropriate for the site (South America Albers for Pacific and Africa Albers for Atlantic). To represent the distribution of birds at each wintering site, we created 25%, 50%, and 75% occupancy contours in GME [40].

We calculated great-circle distances between each pair of valid locations in Program R [35], and subsequently calculated distance per day based on the number of days between locations. Travel distance (km) was defined as the distance travelled during fall migration not including movement during stopover periods, and travel speed (km/d) as the travel distance divided by the days travelled (“travel” days only) during fall migration (as per [44]). Welch’s *t*-test was used to test for differences in travel distance and speed between wintering populations in Program R [35].

## Results

We recovered 38 of 47 (81%) geolocators deployed on Nasaruvaalik Island, Nunavut from 2008 to 2012. Four additional tagged birds were seen at the colony but did not breed, while one bird returned and successfully bred without its tag (92% of tags were re-sighted). After filtering, our dataset contained 6,354 locations (91.8% valid), averaging 177 days per track. Twenty-eight geolocators tracked birds to their wintering site, while eight geolocators confirmed migration direction (Pacific or Atlantic) but failed before arrival to the wintering site. Two geolocators failed during the breeding season and were not included in the analysis (*n* = 36). Ten birds were tracked twice; therefore our data describe the movement of 26 individual birds.

**Fig 2:**
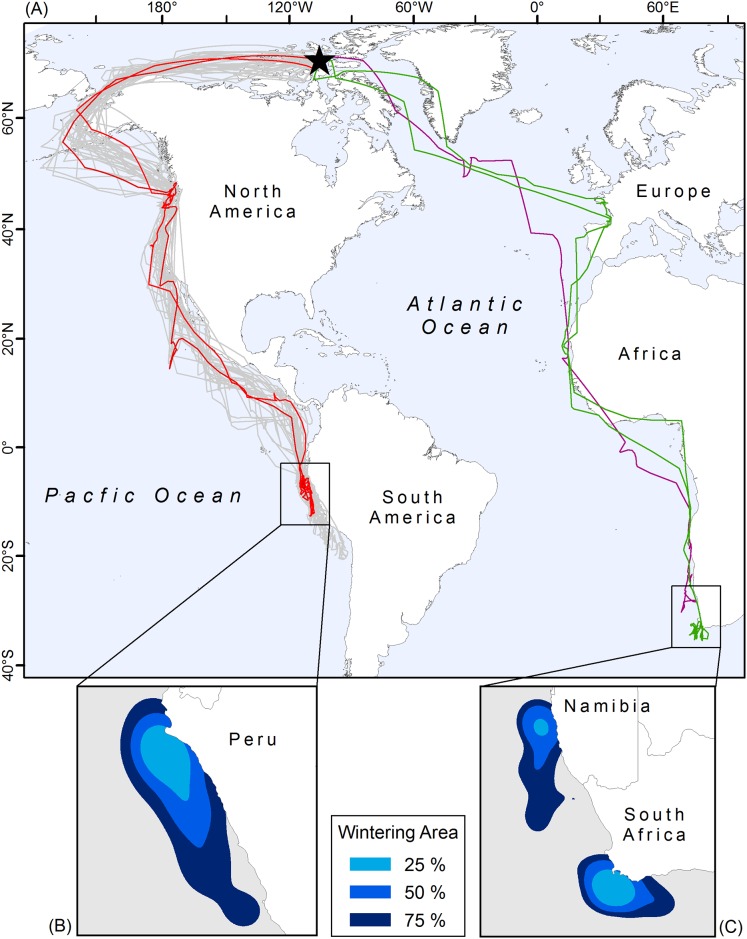
Southbound migration and wintering area of Sabine's gulls (*Xema sabini*) breeding at a site in the Canadian High Arctic. (A) Showing study site (black star), Pacific tracks (*n* = 33, 24 individuals), and Atlantic migrants (*n* = 3, 2 individuals). One breeding pair tracked for two consecutive years spent both non-breeding seasons in different oceans (red = Pacific female, green = Atlantic male). (B) Pacific wintering area, with 25%, 50%, and 75% occupancy contours (*n* = 26). (C) Atlantic wintering area, with 25%, 50%, and 75% occupancy contours (*n* = 2).

Birds breeding on Nasaruvaalik Island disperse to both the Atlantic and the Pacific oceans during the non-breeding season (Fig 2-A). The majority of birds tracked (93%) migrated west to the Pacific wintering site (Fig 2-B), while two of the birds tracked (7%) migrated east to the Atlantic wintering site (Fig 2-C). Remarkably, one pair of Sabine’s gulls (confirmed mates for six seasons 2009–2014) migrated to different oceans for the non-breeding season; the female migrated west to the Pacific (Fig 2-A; red tracks) while the male migrated east to the Atlantic (Fig 2-A; green tracks). This pair of birds was tracked for two consecutive years (Fig 2-A; represented by 2 tracks of the same color for each bird).

Sabine’s gulls showed high wintering site fidelity; all ten birds that were tracked for two years wintered in the same area both years, including one Atlantic wintering bird.

Sabine’s gulls left the breeding site in late August and arrived at the wintering site in early November (Table 1). During fall migration, tagged birds travelled 14,578 km to the Pacific wintering site, and 14,615 km to the Atlantic wintering site, excluding movement during stopover periods (Table 1). Both Pacific and Atlantic birds spent 84 days migrating to the wintering site, flying at a speed of c. 350 km/day on travel days (Table 1).

**Table 1:** Southbound migration details of Sabine’s gulls (*Xema sabini*) tracked with geolocators from the Canadian High Arctic (Nasaruvaalik Island) over three years (2008/09, 2010/11, 2011/12), showing mean value and range (min—max).

	Pacific Migrants	Atlantic Migrants
Migration tracks (*n*)	33	3
Departure from breeding area	18 Aug (5 Aug– 3 Sep)	24 Aug (10 Aug– 1 Sep)
Arrival at wintering area	11 Nov (15 Oct– 6 Dec)	12 Nov (10 Nov– 15 Nov)
Duration of fall migration (d)	84 (58–112)	84 (75–92)
Distance travelled (km)	14,578 (12,711–17,732)	14,615 (12,684–16,545)
Travel speed (km/day)	347 (252–514)	354 (345–362)

There was a statistically significant difference in travel distance between years (travel speed did not differ significantly) as determined by a one-way ANOVA (*F*_2,25_ = 3.4, *p* = .049), however post hoc comparisons using a more conservative Tukey HSD test showed travel distance did not significantly differ among years. When comparing migration metrics between wintering populations, we found no significant difference in travel distance (*t*_1_ = 0.02, *p* > 0.5) or travel speed (*t*_8_ = 0.41, *p* > 0.5) between Pacific and Atlantic migrants.

## Discussion

Here, in the first tracking study of Sabine’s gulls from the North American Arctic, we report that birds from a single colony dispersed to both the Pacific and Atlantic oceans during the non-breeding season. This study confirms a migratory divide for this species in the Nearctic around 96° W. Our work on Sabine’s gulls is one of only a few other studies documenting breeding populations of any species from the Canadian Arctic moving to disjunct wintering areas [12,17]. Because much of the North American Arctic has only relatively recently been exposed after the last glacial period, the colonization and migration patterns of birds breeding there are difficult to interpret; some species show distinct genetic structuring in populations (e.g. northern fulmars *Fulmarus glacialis*; [45]) while others do not (e.g. ivory gulls *Pagophila eburnea*; [46]). Such differences may be attributed to how long these populations were isolated as well as their propensity to colonize newly available habitat following glacial periods. Combined data from several species and studies suggests a zone of transition or overlap between Atlantic and Pacific wintering populations around 100° W in the Canadian Arctic. [11,12,17].

In the High Arctic, migratory divides occur between areas which offer an optimal combination of suitable breeding habitat balanced with a relatively low cost of migration to suitable wintering habitat, considering both the distance to travel as well as the ecological or topographical barriers *en route* [47,48]. Our results show that Sabine’s gulls travelling from Nasaruvaalik Island to either of the two wintering sites used face very similar energetic costs, at least in terms of flying distance, speed, and duration.

Throughout most of their breeding range, Sabine’s gulls prefer low-lying tundra habitat associated with freshwater or tidal marshes [19]. Only a small portion of the global population of Sabine’s gulls breeds in the High Arctic [19], and little is known about Sabine’s gulls breeding in the northernmost part of their range, such as those we studied here. The nearest known major breeding colonies of Sabine’s gulls lie hundreds of kilometers to the southeast and southwest [19,49] of our study site, yet Sabine’s gulls breeding on Nasaruvaalik Island experience higher reproductive success [27] than birds breeding in more typical Low Arctic environments [49]. Consequently we suggest that the birds nesting at Nasaruvaalik Island may represent a relatively recent colonization of particularly favorable habitat by a diverse and distinct population of birds representing the northernmost breeders from both Atlantic and Pacific wintering populations, consistent with the theory that the colonization of suitable breeding habitat may be one of the strongest drivers of range expansion in the High Arctic. Nasaruvaalik Island has been identified as one of the most important breeding sites for a wide variety of ground-nesting seabirds in the Canadian High Arctic on account of several small but highly productive polynyas nearby that provide reliable foraging opportunities when surrounding waters are still completely frozen in the early breeding season [50].

The brief and unpredictable High Arctic breeding season places a high premium on timing arrival at the breeding site to coincide with optimal nesting conditions, and for individuals to arrive in prime breeding condition [8]. Coordination of behavior within pairs during the breeding season (e.g. timing of foraging trips, nest defense) is often pronounced in long-lived seabirds which require biparental care for successful reproduction [51]. Outside the breeding season however, behavior is driven by prey availability, genetics, and/or climate, and mated pairs may winter in the same area because of shared traits rather than coordination of behavior [51]. Our study shows that in rare cases, mated pairs of birds migrate to opposite ocean basins during the winter, returning to the same breeding site without knowing how the schedule of their respective partner is affected by environmental conditions *en route*. Even birds migrating along the same routes and relying on the same cues to time their arrival at breeding sites are susceptible to misjudging local conditions upon arrival [52]. Although some polar seabirds disperse from a single colony to disparate wintering areas [53,54], this appears to be a rare phenomenon, and to our knowledge, our results are the first confirmed example showing divergent migratory pathways between members of a breeding pair of any species. Sabine's gulls form strong multi-year pair bonds [55], and the reproductive costs involved in deferring breeding or finding a new partner if a former mate fails to arrive at the breeding site are considerable, and would presumably be exaggerated in mixed pairs arriving from different directions. While it is difficult to extrapolate beyond the one example we discovered of a mixed pair, the fact that these individuals have bred successfully over four consecutive years suggest that either the conditions at Nasaruvaalik Island are particularly favorable in order to sustain this risky union or this site is far enough north that there may be less variability in the possible timing of nesting, so the breeding window is very small.

Information about how populations are geographically linked throughout the year is lacking for many species of migratory birds [56], including Sabine’s gulls [19]. This research is the first to examine the degree of migratory connectivity in Sabine’s gulls breeding in the Nearctic, and shows that birds breeding on Nasaruvaalik Island exhibit somewhat diffuse migratory connectivity due to mixed wintering area preference. Ultimately, this study provides new insight into the migration ecology and behavior of Arctic breeding migrants. Limited areas of suitable breeding habitat within the High Arctic attract and sustain colonies of birds nesting at the limits of their range. At high latitudes, breeding colonies that lie relatively equidistant from suitable winter habitat may consist of individuals from different wintering populations, as shown in this study. The reproductive disadvantages of increased variability in timing of migration and arrival within a single breeding population at such mixed colonies may be offset by exceptionally favorable breeding conditions at specific sites such as Nasaruvaalik Island.
